# Metacognitive Effort Regulation across Cultures

**DOI:** 10.3390/jintelligence11090171

**Published:** 2023-08-23

**Authors:** Rakefet Ackerman, Avital Binah-Pollak, Tirza Lauterman

**Affiliations:** Technion—Israel Institute of Technology, Haifa 3200003, Israel; avitalbp@technion.ac.il (A.B.-P.); tirza2502@gmail.com (T.L.)

**Keywords:** metacognition, meta-reasoning, problem-solving, mental effort regulation, monitoring accuracy, mental effort stopping rules, non-verbal problems

## Abstract

Success in cognitive tasks is associated with effort regulation and motivation. We employed the meta-reasoning approach to investigate metacognitive monitoring accuracy and effort regulation in problem solving across cultures. Adults from China, from Israel, and from Europe and North America (for simplicity: “Western countries”) solved nonverbal problems and rated their confidence in their answers. The task involved identifying geometric shapes within silhouettes and, thus, required overcoming interference from holistic processing. The Western group displayed the worst monitoring accuracy, with both the highest overconfidence and poorest resolution (discrimination in confidence between the correct and wrong solutions). The Israeli group resembled the Western group in many respects but exhibited better monitoring accuracy. The Chinese group invested the most time and achieved the best success rates, demonstrating exceptional motivation and determination to succeed. However, their efficiency suffered as they correctly solved the fewest problems per minute of work. Effort regulation analysis based on the Diminishing Criterion Model revealed distinct patterns: the Western participants invested the least amount of time regardless of item difficulty and the Israelis invested more time only when addressing the hardest items. The Chinese group allocated more time throughout but particularly in moderate to difficult items, hinting at their strategic determination to overcome the challenge. Understanding cultural differences in metacognitive processes carries implications for theory (e.g., motivational factors) and practice (e.g., international teams, education). The present findings can serve as a foundation for future research in these and other domains.

## 1. Introduction

The mental operations involved in addressing challenging thinking tasks (e.g., solving logical problems) require the activation of cognitive mechanisms, such as searching for prior relevant knowledge, processing the given information, choosing a response, and more (Funke et al. 2018). These cognitive processes are necessarily accompanied by the metacognitive regulation of mental effort, expressed through actions like setting a goal, monitoring progress toward that goal, and allocating one’s time accordingly (see Fiedler et al. 2019 for a review). Extensive theoretical frameworks deal with the associations between self-regulation, motivation, and cognitive achievement (Bandura 1986; Sundre and Kitsantas 2004). These theories offer substantial evidence that (a) self-regulation and motivation are interconnected processes (Pintrich and De Groot 1990) and (b) both factors are positively linked to higher cognitive achievement (see Schunk and Zimmerman 2023 for a review).

The metareasoning framework put forward by Ackerman and Thompson (2017) describes a set of metacognitive monitoring and control processes specifically relevant to reasoning, problem-solving, and decision-making tasks. This framework has increasingly been used to guide investigations into factors affecting monitoring accuracy and the allocation of effort in these contexts (see Fiedler et al. 2019). Most research under this framework has been focused on delineating generalized principles common to problem solvers in general while differing between tasks (e.g., knowledge domain, Dentakos et al. 2019) or items (e.g., easier vs. harder items, e.g., Bajšanski et al. 2019; see Ackerman 2019 for a review). Some, though not much, of the metareasoning literature deals with individual differences (e.g., Kleitman et al. 2019; Law et al. 2022; Teovanović 2019). The literature addressing culture-dependent metacognitive differences is particularly scant.

A recent theoretical framework, the Cultural Origins of Metacognition (Heyes et al. 2020), suggests that culture-dependent facets of metacognition are determined by the extent to which cultures place emphasis on understanding the mental states of the self and others. By this framework, strong metacognitive skills may benefit not only the individuals possessing them but also social groups that make decisions and work together. Consequently, the metacognitive processes of individuals are expected to exhibit variations across cultures with distinct social characteristics. So far, this conjecture has been supported with perceptual tasks (Moore et al. 2018; Van der Plas et al. 2022), general knowledge questions (J. W. Lee et al. 1995; Whitcomb et al. 1995), and diagnoses of fictional diseases (Yates et al. 1998). However, recent metareasoning advancements provide concepts and tools for examining, in more depth than before, the effectiveness of effort regulation.

In the present study, we examined cultural differences in monitoring accuracy and self-regulation strategies when solving challenging problems by comparing three cultural groups: Chinese undergraduates, Israeli undergraduates, and a group sampled from the general public (but including university students) in Europe and North America (hereafter, for convenience: Western countries). We used nonverbal problems, which are particularly suited to cross-cultural comparison as they minimize the effects of semantic differences between languages. Within the metareasoning research domain, as in metacognitive research, generally, most studies have employed verbal tasks (e.g., the Cognitive Reflection Test, De Neys et al. 2013; syllogisms, Trippas et al. 2017; the Remote Associate Task, Topolinski and Strack 2009). Thus, the present study goes beyond the culture-based analyses as it adds to our understanding of monitoring accuracy and effort regulation when facing nonverbal problems.

In this study, all groups solved the same set of nonverbal problems based on geometric shapes. The task did not require any formal prior knowledge. Participants rated their confidence immediately after solving each problem. In addition, we measured response success rate and solving time.

## 2. Culture-Dependent Metacognitive Monitoring Accuracy

Reliable (i.e., accurate) metacognitive monitoring is crucial to effective learning and general cognitive performance as this is the basis on which people choose between different control actions—e.g., whether to continue or stop investing thinking effort, to seek help or to provide an answer vs. an “I don’t know” response (Fiedler et al. 2019). For the present cross-cultural comparison, we employed two primary measures of monitoring accuracy: resolution and calibration (see Ackerman et al. 2016). Both measures associate the success and confidence judgments provided by each participant. *Resolution* refers to the relative accuracy of confidence judgments in terms of individual items and reflects the extent to which the participant distinguishes between his/her own correct and incorrect answers (e.g., de Bruin et al. 2011; Thiede et al. 2010). *Calibration* refers to the absolute difference between each participant’s mean confidence in his/her answers and a participant’s success rate across all task items. Calibration, more commonly, is found to capture a tendency towards overconfidence, rather than underconfidence, which is relatively rare (e.g., Sidi et al. 2017).

Thus far, only limited findings describe culture-dependent metacognitive characteristics. The traditional monitoring accuracy measure used in this domain—typically with general knowledge questions—has been calibration (J. W. Lee et al. 1995; Whitcomb et al. 1995). These findings point to culture-dependent variations in calibration. However, the results have not been straightforward. Some studies have found that individuals from certain Asian cultures (e.g., the Chinese) tend to display significantly more overconfidence compared to individuals from other cultures (Moore et al. 2018; Yates et al. 1998). However, Stankov and Lee (2014), using a numerical task, found less overconfidence in participants from East Asian nations, including China, compared to Western countries. They suggest that differences in calibration result chiefly from differences in success rates and that confidence judgments are actually similar across different parts of the world. In line with that speculation, Lundeberg et al. (2000) found that Taiwanese students exhibited lower academic achievement and higher overconfidence compared to students from Israel, the Netherlands, and the United States. Morony et al. (2013) found that students around 15 years old from some European counties scored lower and were more overconfident when solving mathematics problems than students of the same age from East Asian countries. This phenomenon aligns with the broadly replicated Dunning–Kruger effect, whereby individuals who lack skill or knowledge are “doubly cursed” because they also insufficiently acknowledge their deficiency (Dunning et al. 2003). This effect explains the contradictory calibration results as stemming from variations in performance rather than from cultural differences in metacognitive processes.

Regarding resolution, recently, Van der Plas et al. (2022) found that Chinese participants showed better resolution in perceptual decision-making tasks compared to U.K. participants. Lundeberg et al. (2000) also found that Taiwanese students had better resolution than Israeli, Dutch, and U.S. students in their judgments of their academic scores. These differences in monitoring accuracy across cultures provide initial hints of metacognitive differences yet to be delineated.

Metacognitive research involving reading-comprehension tasks has shown associations between the depth of processing and resolution (see Prinz et al. 2020 for a meta-analysis). For instance, participants achieved better resolution when they were guided to write keywords that reflected the gist, compared with spontaneous learning (de Bruin et al. 2011). The contribution of depth of processing to calibration is less commonly documented. However, applying a similar keyword method, Lauterman and Ackerman (2014) found improved calibration when participants learned in a computerized environment, even to the extent of canceling out screen inferiority—the disadvantage of working in a computerized environment relative to performing the same tasks on paper (see also Sidi et al. 2017). It is not yet known how depth of processing is associated with resolution and calibration in problem-solving tasks and how these are connected to the allocation of time.

In the present study, we focused on the allocation of thinking time. Previous research in both memory and problem solving suggests that high motivation to succeed leads to more time investment in more challenging items, but not in easier ones, within the item pool (Ackerman 2014; Undorf and Ackerman 2017). This strategic behavior is rational because processing challenging items can benefit from extra time investment while easy items are processed quickly with little room for motivational variations. We hypothesized that more thinking time would improve resolution and calibration, just as manipulations meant to trigger in-depth processing were shown to affect monitoring accuracy in reading comprehension. We examined whether participants who invest more time do so strategically, devoting more time to items that carry hope for improvement compared with both easy items and those that are much more difficult, potentially beyond the person’s capacity to solve (see also Ackerman and Levontin, forthcoming).

## 3. Control: Goal Setting and Effort Regulation

According to the classical models of metacognition known as *discrepancy reduction models*, when people learn (e.g., memorize a list of words), they first set a goal—a desired knowledge level that will bring satisfaction to their learning. Then, they invest time in a goal-driven manner until their metacognitive judgment, regarding their knowledge, reaches this preset goal (Dunlosky and Hertzog 1998; Nelson and Narens 1990; see illustration in Ackerman and Goldsmith 2011). Adapting this principle to problem solving and decision making, metareasoning research has shown that as people mull over potential answer options, they invest effort in accumulating evidence that increases their confidence in the option currently being considered (see also Evans 2006; Glickman et al. 2022; Pleskac and Busemeyer 2010; Yeung and Summerfield 2012).

According to the discrepancy reduction models, the time people invest in finding a solution increases as the problems become more difficult. These models also predict that with increased effort investment in a particular task item, confidence levels should rise until they meet or exceed the solver’s preset confidence goal. However, these models do not address cases in which confidence does not rise as more time is invested, either because one lacks the knowledge needed to solve the problem or simply because no acceptable solution comes to mind (Ackerman and Morsanyi 2023). Indeed, numerous studies have shown that after lengthy thinking time, people tend to provide low confidence judgments, resulting in persistent inverse relationships between time and confidence (e.g., Koriat et al. 2006).

**Figure 1:**
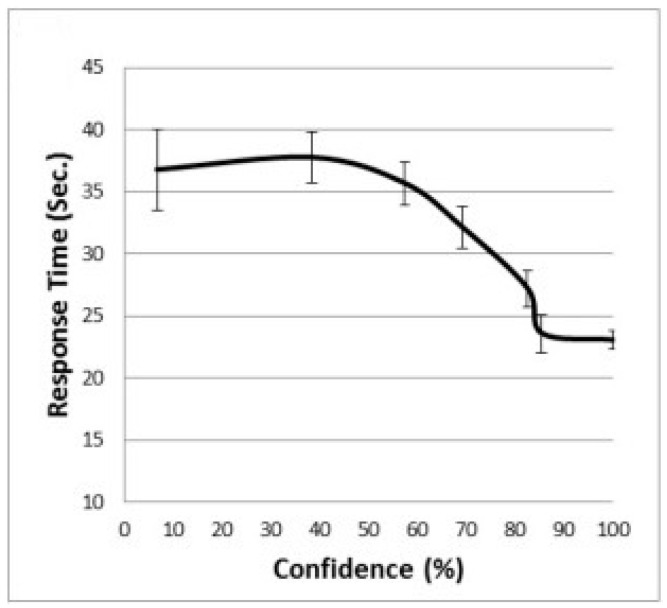
Example of response time as a function of confidence in Raven’s matrices. Error bars represent standard errors of the means. The figure was adapted from Ackerman et al. (2020a).

In an attempt to explain why people give up on problems with low confidence after lengthy thinking, Ackerman (2014) suggested the *Diminishing Criterion Model* (DCM). According to the DCM, people’s goals are not static but, rather, shift downward as more time is invested in a given task item. Thus, the more time is invested in processing the problem, the more the respondent becomes willing to compromise—to provide a response with less confidence than would have been required for a response that came to mind quickly. Moreover, the DCM includes a time limit—a length of time that the solver sees as reasonable for addressing each task item. When no solution comes to mind within this period, solvers provide the best solution they can come up with, even if it is accompanied by lower confidence than they would originally have preferred. Response time, thus, reaches a plateau at the time limit for the items with the lowest and moderately low confidence levels (see Figure 1 for an example). According to the DCM, combining these two stopping rules produces the curvilinear confidence–response time (RT) association often seen in empirical studies.

Support for this model has been found across diverse tasks, including memorizing words, solving problems of several types, web searching, and using an application for merging databases (Ackerman et al. 2019, 2020a; Undorf and Ackerman 2017). Studies have also found that the stopping rules under the DCM are sensitive to various factors, including motivation (Ackerman 2014; Undorf and Ackerman 2017), time pressure (Ackerman 2014), and a priming manipulation aimed at inducing a growth vs. fixed mindset (i.e., beliefs about whether intelligence can be developed; Ackerman and Levontin, forthcoming). However, no such detailed analysis of the DCM’s stopping rules has been conducted, as of yet, in the context of cultural differences.

As suggested, concerning other individual differences (e.g., math anxiety, Morsanyi et al. 2019), culture-dependent differences in effort regulation might stem from any of the components in the effort regulation process described above. These include potential differences in initial goal setting, the pace at which confidence rises with invested effort, the slope at which the confidence criterion shifts downward, the solver’s internal time limit, or any combination of these. They could also stem from the educational transfer of attitudes toward working under different time frames (Li 2012).

There is some evidence for differences between East Asian and Western cultures in motivational factors, particularly in a tendency towards promotion vs. prevention motivation. People with a promotion focus are driven by the gains that are expected to follow success while people with a prevention focus aim mainly to meet their responsibilities, avoid failure, and stay safe (Halvorson and Higgins 2013). Typically, East Asians rank higher in prevention motivation while culturally Western individuals rank higher in promotion motivation (Kurman and Hui 2011; Zusho et al. 2005). Scholars who have examined the reasons for East Asians’ higher prevention focus point to factors such as the high levels of academic stress experienced by students in countries such as China, where education is key to achieving upward social mobility and, thus, meeting the criteria for university admission is crucial (Anagnost 2004; Dello-Iacovo 2009; Kipnis 2019; Zhao et al. 2015). Relatedly, people from East Asian cultures tend to be high in uncertainty avoidance, meaning they become anxious when faced with unpredictable and unstructured situations (House et al. 2004). By contrast, Israeli culture is considered to be low in uncertainty avoidance (Erez and Nouri 2010) while the European population is more heterogenous (Sánchez-Franco et al. 2009).

In East Asia, effort investment in cognitive tasks (e.g., learning) is considered exceptionally important (Li 2004, 2006). Indeed, studies have shown that East Asian students tend to attribute success and failure in educational tasks to effort investment while Western students are more likely to credit (or blame) personal (in)ability (Stevenson and Stigler 1992). This difference resonates with the literature on the costs of investing mental effort (Shenhav et al. 2017) and, particularly, the effects of the differences between a growth and fixed mindset regarding intelligence (Dweck et al. 1995). People with a growth mindset expect to develop their intelligence by facing cognitive challenges while people tending towards a fixed mindset interpret failure as testifying to innate inability. Thus, effort investment when facing cognitive tasks can be interpreted as a sign either that the task is important for one’s mental development (growth) or that any further effort is doomed to failure (fixed; see Oyserman et al. 2015). Indeed, previous metacognitive research has associated a growth mindset with more strategic regulation of effort than a fixed mindset (Ackerman and Levontin, forthcoming; Bae et al. 2021; Miele et al. 2011). In this study, we investigated how cultural variations, including differences in mindset, relate to variations in monitoring accuracy and self-regulation strategies when solving challenging problems.

## 4. The Present Study

Given the indications of cultural differences in motivational factors found in prior research, the cultures considered in this study were expected to differ in how people set and adhere to their stopping rules. With respect to the DCM, the cultures were expected to differ particularly in the time participants were willing to invest in each task item before moving on to the next one. Specifically, high determination to succeed, known to characterize East Asians, was expected to shift their time limit upwards, leading them to invest more time in solving the problems. The practical question derived from this prediction is whether their outcomes benefit from this additional time investment. For delving into this question further, we examined whether this shift is similar across all items or depended on how challenging each one was. We expected the difference to manifest in the challenging items in the pool more than in the easier ones. A question of interest was whether East Asian participants would invest more time in the items they experienced as the most difficult or even impossible to solve (the lowest end of the confidence scale) or mainly in those they saw as carrying hope for improvement with additional thinking time (pushing upwards the time invested mainly in items that ended up with intermediate confidence levels). When considering cultural differences in monitoring accuracy, we expected that the increased time investment by East Asian participants would benefit both resolution and calibration due to the association between time investment and depth of processing reviewed above.

To test these hypotheses, we conducted a comparative analysis between Chinese undergraduates, Israeli undergraduates, and a group sampled from the general public in Europe and North America. We hypothesized that Chinese students would display a greater willingness to invest effort in problem-solving attempts compared to the other two groups. Furthermore, they were expected to refrain from providing answers with low confidence, leading to higher overall confidence levels compared to the other groups. We further anticipated that Chinese students would exhibit better resolution and calibration. Additional hypotheses related specifically to the task we used are detailed below.

**Figure 2:**
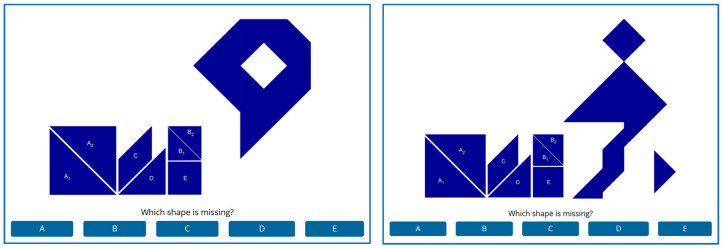
Examples of the Missing Tan Task (MTT).

We used the Missing Tan Task (MTT), a nonverbal task recently introduced by Ackerman (2023). The MTT is a multiple-choice variation of the Tangram game, originally developed in China (https://en.wikipedia.org/wiki/Tangram (accessed on 21 August 2023)). In the original task, silhouettes are generated from all seven geometric pieces presented in the legend of Figure 2. In the MTT version, each silhouette is generated by six pieces out of the seven and the task is to identify which piece is missing. See examples in Figure 2.

The original Tangram task has been used for teaching geometry and for training spatial abilities in educational contexts (Bohning and Althouse 1997; J. Lee et al. 2009). It has also been used in neuroimaging studies to offer insights into spatial thinking in the frontoparietal brain networks and parts of the prefrontal cortex involved in cognitive functions, such as attention, working memory, and cognitive control (e.g., Hu et al. 2019).

Our three samples were selected so as to ensure a comparison between culturally distinct groups. They included two groups of undergraduates, from China and Israel, and a group from the general public in Europe and North America (our “Western” sample). The Western sample comprised adults drawn from the participant pool of Prolific.ac, with no restrictions beyond self-reported English knowledge and an age of 18 or above. This platform is known to provide high-quality data that replicate lab findings (Peer et al. 2017, 2022). Given that we took the sample from an online platform, by definition, all members of the Western group had some proficiency in doing online tasks. Notably, of the sample we obtained, more than half were university students.

Both sets of undergraduate participants were students in prestigious programs. Clearly, the general public—even in a sample including some students—differs in many respects from undergraduates who have been accepted into selective programs. Ackerman (2023), using a different geometric task, found better success and calibration and worse efficiency among undergraduates in samples from the very same Western and Israeli populations as used here. However, Ackerman’s results showed equivalences between these groups in confidence, calibration, response time, and resolution.

With respect to the Chinese sample, two different lines of reasoning point in two different directions. First, prior research suggests that members of Eastern cultures solve problems in a more holistic manner than members of Western cultures, meaning that they tend to pay more attention to the whole picture while Westerners are more likely to focus on the details (Ji et al. 2000; Miyamoto et al. 2006; Nisbett et al. 2001). Given that the MTT requires decomposing an image into its parts, members of the Chinese sample, to the degree that they indeed take a more holistic approach, might be disadvantaged. However, MTT items are not identical in the degree to which component shapes are concealed within the whole. For instance, the right-hand example in Figure 2 is more spread out and includes clearly identifiable shapes while the left-hand example is highly compact, making it harder to isolate individual shapes. This aspect of the stimuli allows a unique opportunity to examine the effects of holistic perception on performance. Following the review above, Chinese participants were expected to be highly motivated to solve the problems correctly. In terms of effort regulation under the DCM, allowing more time to solve each problem was expected to enable them to overcome a cultural tendency toward holistic perception.

In terms of performance metrics, Ackerman (2023) found that while high motivation indeed increased success in the task, it also harmed efficiency (correct solutions per minute of work) because the proportional increase in time was much larger (300%) than the increase in success (30%). In light of these metacognitive analyses, we expected the Chinese group to solve more task items correctly than the other two groups but to have lower efficiency because of their disproportionally long time investment.

As background information for examining our main hypotheses, we gathered data on the dispositions of the three groups in terms of time management, need for cognition (NFC), actively open-minded thinking (AOT), and mindset regarding intelligence (fixed vs. growth). Need for cognition is a stable personality trait reflecting a tendency to engage in and enjoy effortful cognitive activity (Cacioppo et al. 1996). Given the strong prevention focus and high uncertainty avoidance previously found in East Asian cultures (Hofstede et al. 2010; Kurman and Hui 2011; Zusho et al. 2005), we can expect the Chinese sample to score relatively high in need for cognition. By the same token, we can expect the Chinese sample to score relatively low in actively open-minded thinking, defined broadly as a willingness to consider alternative opinions or new ideas, and to postpone closure (Ku and Ho 2010). As for mindset regarding intelligence, by the same reasoning adduced above, we expected members of the Chinese sample to display a more fixed mindset; although, the expected extra determination to succeed while facing a challenging task could stand in contrast with this expectation and be more in line with a tendency toward a growth mindset. For details of the measures used, see Materials, below. Several attention-verification questions were used for screening (see under Participants, below).

## 5. Method

### 5.1. Participants

A priori power analysis with G*Power indicated that a total of 119 participants would be sufficient enough to achieve statistical power (*β* = 0.95) to detect a moderate effect size (*f*^2^ = 0.15) for a multiple-regression analysis with three predictors: one between-participants variable with three groups and two within-participants variables (Faul et al. 2007). Thus, the analysis called for at least forty participants in each group. However, given that this is the first study dealing with individual differences in the DCM pattern across cultures, we aimed to provide more precise estimates of the population parameters. We, therefore, recruited a minimum of twice as many participants as were needed for each group.

Before the screening, the Chinese group included 81 students from the Guangdong Technion—Israel Institute of Technology (GTIIT), a higher education institution established jointly by the Technion and Shantou Universities in Guangdong province, China. The Israeli group included 149 students from the Technion—Israel Institute of Technology, Israel. Both the Chinese and Israeli students were studying towards Engineering degrees and had met high admission requirements. The Western group was recruited via the Prolific Academic platform and included 85 participants above the age of 18 from Europe and North America, including 45 (58%) university students.

Exclusion criteria were defined a priori as displaying at least two indications of low attention among the following: investing more or less than 2SD above or below the sample’s mean response time; switching away from the task browser window for more than 25% of the measured time; providing correct answers at a rate less than chance (20%); lack of variability in confidence ratings (e.g., all 60%); and incorrectly answering an attention-verification question that followed the self-report questions (see Materials). In total, 7 of the Chinese participants (9%) were excluded, leaving 74 participants in the Chinese sample (*M*age = 20.2 *SD* = 0.9, 47% female). In addition, 6 participants (4%) were excluded from the Israeli group, leaving 143 participants (*M*age = 24.6, *SD* = 2.8, 46% female), and 8 participants (9%) were excluded from the Western group, thus leaving 77 participants in this sample (*M*age = 25.3, *SD* = 6.1, 49% female).

### 5.2. Materials and Measures

We used the Missing Tan Task (MTT) introduced by Ackerman (2023). See the description above and examples in Figure 2. We used 31 MTT items, including 1 for training.

Self-report questions were presented after the task. Four sets of items concerned dispositional factors: time management, need for cognition, open-minded thinking, and mindset with respect to intelligence (fixed vs. growth). Time management (three items) was assessed via the time management scale of the Individual Learning Profile (ILP, Pulford and Sohal 2006); a sample item read “Do you typically deliver work on time?”. Need for cognition (NFC; three items) was measured by the Need for Cognition Scale of Cacioppo et al. (1996); e.g., “Thinking is my idea of fun”. Another three items measured Actively open-minded thinking (AOT, Haran et al. 2013); e.g., “Allowing oneself to be convinced by an opposing argument is a sign of good character”. Mindset regarding intelligence, fixed vs. growth, was measured with four items drawn from the work of Dweck et al. (1995); a sample item read “You can learn new things, but you cannot really change your basic intelligence” (high values indicate a fixed mindset). After the dispositional items, respondents were asked one question about their perceptions of the task (“How challenging was the task?”) and one question about their experience with games and puzzles (“How often do you solve puzzles or play thought-provoking games?”). All questions were in Likert format, with a scale ranging from “Not at all” (1) to “Very much so” (7). Some questions were reverse-scored. An additional question used as an attention check asked participants to wait four seconds and then respond with 2 on a 1–7 scale.

### 5.3. Procedure

The study procedure was approved by Technion’s ethics committee (2022-024). All participants signed an informed consent declaration at the outset of this study, before proceeding to the task.

All participants performed the experimental task online and unsupervised. The experiment was administered in English for the Western group and in Mandarin Chinese and Hebrew for the Chinese and Israeli groups, respectively. The task began with general instructions, including the expected time it should take to complete all problems and the procedure for providing confidence ratings (see below). The instructions concluded with an example, followed by an explanation of the solution.

The problems were presented in a random order generated for each participant. For each problem, we measured response time (RT) from the moment the problem was presented until the moment participants clicked their chosen option. Clicking the chosen option also activated a confidence scale on the same screen. The scale ranged from “Definitely wrong” (0%) to “Definitely correct” (100%). Participants were instructed to rate their confidence by moving a slider to the appropriate spot on the scale immediately after providing each solution. The chosen option could not be changed when the confidence rating scale was active. Clicking “Next” after rating confidence initiated the presentation of the next problem. After the main task, participants answered the self-report questions.

### 5.4. Analysis Plan

First, we documented dispositional differences between the groups based on the self-report questions. We then examined the classic dependent variables in metacognitive research (e.g., Ackerman et al. 2016). Specifically, for each participant, we calculated the mean success rate, response time, confidence, overconfidence (mean success rate subtracted from mean confidence), and resolution (within-participant gamma correlation between confidence and success for each item).

To examine effort regulation under the DCM, for each group, we generated a hierarchical regression model predicting RT by confidence and confidence squared. The linear predictor of confidence denotes the overall slope of the confidence–RT relationship, with a negative regression coefficient indicating an inverse linear confidence–RT association, as found in numerous studies (e.g., Glöckner and Betsch 2008; Koriat et al. 2006; Thompson et al. 2013). The quadratic predictor of confidence squared yields the curvilinear confidence–RT relationship, with a negative coefficient indicating an inverted U-shaped relationship, a positive coefficient indicating a U-shaped relationship, and an insignificant coefficient indicating no curvilinearity. An inverted U-shaped relationship is the unique signature of the DCM and it reflects where people position their stopping rules when allocating time to each task item. Differing degrees of curvature, expressed in different coefficients of confidence squared, imply differing degrees of perseverance.

Finally, to examine the interference of holistic processing, we used the area of the imagined rectangle that encompasses each silhouette as a measure of the silhouette’s compactness and, therefore, the degree to which different shapes stand out from the rest. Figure 2 presents two examples. The figure on the left is more compact; so, the individual shapes are difficult to distinguish. The figure on the right is more spread out, with spaces between some shapes that make them easier to identify; this also increases the area of the imagined rectangle surrounding the silhouette. Thus, we expected an overall positive correlation between the size of the areas around each silhouette and success. However, our main interests were cultural differences and effort regulation. From this perspective, we expected this positive correlation between the surrounding area and success to be strongest for the Chinese participants, who were assumed to favor holistic processing. At the same time, we also considered the possibility that the Chinese participants would compensate for the challenge inherent in processing highly compact silhouettes by investing more time than the other groups. In this case, their success rates would likely be higher than for the other groups; but, the overall negative correlation between the surrounding area and response time would be stronger for the Chinese group than for the others.

## 6. Results and Discussion

Table 1 presents descriptive statistics. All measures showed variability, ruling out ceiling or floor effects, all *p*s < .001.

Starting with the self-report questions that followed the main task, there were significant differences between the three groups in all questionnaires. Relative to the Western group drawn from the general public, both undergraduate groups had less experience with thought-provoking games and a higher need for cognition. Interestingly, all three groups differed significantly only in self-reported time management, with the Chinese group raked the lowest and the Israelis the highest. As time management is the focus of this study, the associations between this variable and participants’ actual management of time in the task are, thus, of particular interest. The rest of the self-reports showed equivalences between the Western group and the Israeli sample; meanwhile, the Chinese sample stood out. As expected, the Chinese group scored the lowest in open-mindedness, lowest in time management, and highest in need for cognition; they also had a greater tendency towards a fixed mindset relative to the others.

In terms of the objective measures, as expected, the Chinese undergraduates invested significantly more time than the other groups, to the extent of investing more than twice (210%) the time put in by the Western participants. Indeed, this lengthy time investment yielded the best success in solving the problems. However, the relative success benefit (53%) came with significantly lower efficiency. The Israeli undergraduates did not significantly differ from the Western group in all objective measures but tended slightly towards resembling the Chinese group.

A planned-contrast analysis comparing efficiency between the Western general public and the Chinese and Israeli undergraduates in highly selective university programs revealed the surprising result that the members of the Western group were the most efficient, *F*(1, 292) = 10.39, *MSE* = 0.282, *p* = .001, *η*_p_^2^ = 0.034. This finding highlights the importance of in-depth analyses for exposing the practical implications of performance outcomes. The metacognitive analyses below shed more light on this finding.

### 6.1. Metacognitive Monitoring

With respect to metacognitive judgments, the Chinese undergraduates were the most confident in their answers. Given that their success rates were also the highest, this high confidence was justified. Indeed, the calibration of the Chinese undergraduates did not significantly differ from that of either of the other groups. The Western group from the general public showed more overconfidence than the Israeli undergraduates. Unlike calibration, resolution differed significantly between all three groups, with the Chinese students exhibiting the highest resolution and the Western group the lowest.

### 6.2. Metacognitive Control: Goal Setting and Effort Regulation

**Figure 3:**
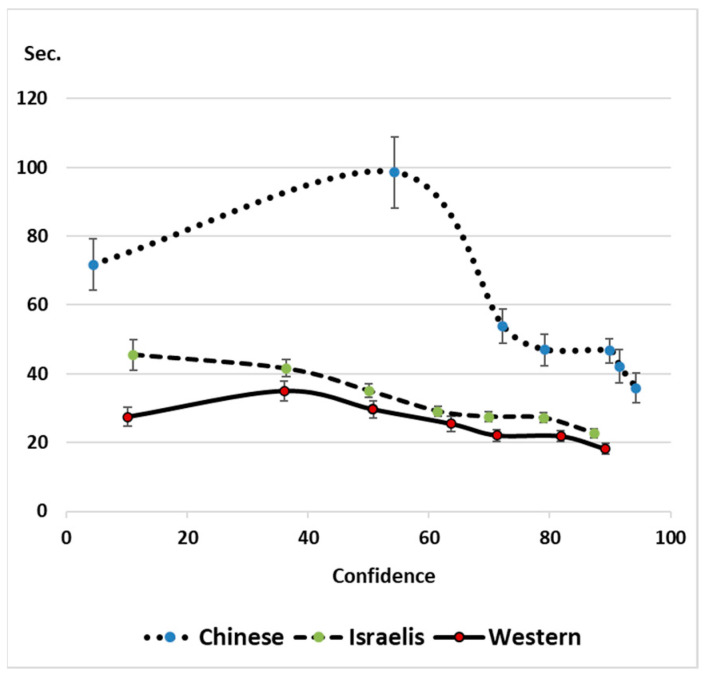
Effort regulation, as predicted by the Diminishing Criterion Model (Ackerman 2014). *Note*. Means of confidence and response time (s) for the seven equal confidence bins of each participant.

We turn next to the DCM pattern representing participants’ stopping rules, based on the intra-participant confidence–RT curvilinear association. To present the results graphically, we divided the confidence judgments of each participant into seven approximately equal bins and calculated the mean RT for each confidence level. Panel A of Figure 3 was generated based on a scatter plot, meaning that both the X and Y axes show meaningful information. The *X*-axis presents the spread of the confidence ratings while the *Y*-axis presents the spread of the response times. In line with the DCM, and in accord with robust prior findings, Figure 3 reveals curvilinear confidence–RT associations for all three groups (Ackerman et al. 2020a; see Figure 1 above). First, the figure clarifies that all groups used the entire confidence scale. Second, the figure sheds light on the differences in time allocation between the groups. The Israeli students invested more time than the Western participants only in the problems that ended with the least confidence. This is the pattern found in previous studies when high incentives were associated with success in the task (Ackerman 2014; Undorf and Ackerman 2017).

The Chinese group showed a somewhat wider range of confidence ratings, as well as a wider range of response times. They also invested more time across the confidence scale, even in their quickest responses, and their confidence ratings were more extreme, with relatively few items having intermediate levels of confidence. Notably, those problems at the middle range of the confidence levels took the most time, showed the largest variability (error bars), and most strongly affected the pattern of effort regulation. This effort regulation pattern is substantially different from that displayed by the other groups.

To statistically compare the confidence–RT associations, we fitted a multilevel regression model using the R packages lme4 and lmerTest (Bates et al. 2015; Kuznetsova et al. 2015). Participants and task items were included as crossed random effects, meaning they were both considered separate random intercepts in the model. Table 2 presents the regression results.

Both the linear component of confidence and the confidence squared component showed the typical curvilinear confidence–RT pattern predicted by the DCM for all three groups. To test for differences in slope and curvilinearity, we used a single regression model that included group and group-interaction terms for confidence and confidence squared as predictors of RT. First, the Chinese and Israeli groups were each compared to the Western group. The significant interactions of the Chinese group with confidence (*b* = −0.71, *t*(8710) = 6.14, *p* < .0001) and confidence squared (*b* = −1.20, *t*(8710) = 3.54, *p* = .0003) revealed that the Chinese group’s negative slope was steeper and curvier than that of the Western group. There was no significant interaction of the Israeli group with confidence (*t*(8710) = 0.59, *p* = .55) or with confidence squared (*t*(8710) = 0.61, *p* = .54), pointing to a similar slope and curvature between the Israeli and Western groups. Comparing the Israeli group to the Chinese undergraduates revealed significant interactions with confidence (*b* = 0.65, *t*(8710) = 6.13, *p* < .0001) and confidence squared (*b* = 1.37, *t*(8710) = 4.42, *p* < .0001), indicating that the Chinese group’s negative slope was steeper and curvier than that of the Israeli group as well. These results point to a unique strategic behavior in the Chinese group that sheds light on the lower efficiency of this group relative to the other two groups. We elaborate on this finding in the General Discussion section.

### 6.3. Interference of Holistic Processing

**Figure 4:**
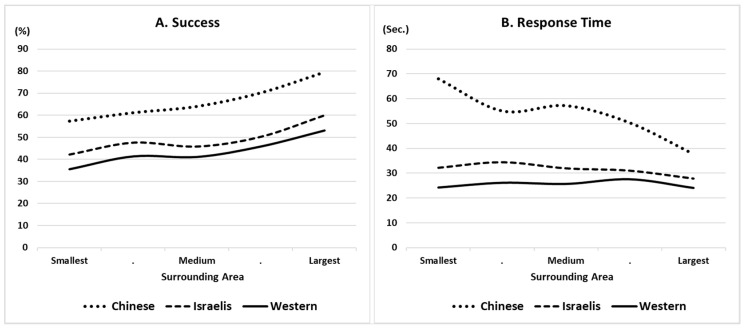
Success (**A**) and response time (**B**) by silhouettes’ surrounding areas.

As mentioned above, in the MTT, the larger the surrounding area of a silhouette (i.e., the less compact the silhouette), the easier the task. This is because in less-compact silhouettes, identifying certain shapes becomes trivial, thereby reducing the number of options to consider as the missing shape. To shed light on this aspect of our study, we examined correlations between the surrounding area and both success and RT for each participant and compared the groups. The results are presented in Table 1 and are displayed graphically in Figure 4. The figure was drawn by splitting the shapes into five bins for surrounding area and calculating the means for success and RT.

Figure 4 shows clearly that the larger the surrounding area, the easier the task, across the cultures. It is also evident that the Chinese group succeeded in solving more problems correctly, regardless of the surrounding area. However, the results in Table 1 indicate that the correlation between area and success was stronger for the Chinese group than for the Israeli group. The pattern in the Western sample was noisier than in the other two groups and did not differ significantly from either.

The pattern for response time shows a stronger distinction between the Chinese sample and the others. It clarifies that the Chinese participants invested most of their time in the hardest items, which were highly compact, while the Western and Israeli participants invested similar amounts of time, regardless of the surrounding area. These findings support the interference of holistic processing, suggesting that the Chinese participants needed extra time to disengage from their natural tendency toward holistic perception when working on the more compact silhouettes. This effort investment points to strong motivation in the face of the challenge; but, it also implies that the more compact silhouettes were a central source for their lower efficiency.

## 7. General Discussion

Metareasoning research has looked at individual differences before but to only a limited extent. Previous research has focused largely on individual differences among participants from a single culture (e.g., Law et al. 2022). In the present study, we used a nonverbal problem-solving task and employed state-of-the-art measures developed in recent years in the metareasoning domain to allow an initial peek into sources for differences in success and efficiency across populations. Specifically, we compared a sample from the general public in Western nations (Europe and North America) to Chinese and Israeli undergraduate groups.

As expected based on cultural differences in the motivation to succeed when solving problems (e.g., Hoshino-Browne 2012; Li 2012), the Chinese group indeed invested more time and had higher success rates than the others. However, metacognitive analyses based on measures of response time, confidence, and success show that this determination comes at a cost in terms of efficiency. Using the metacognitive approach involves collecting RTs and confidence ratings and analyzing within-participant associations between success and these two measures. This approach is highly effective in exposing sources for the found differences in objective measures.

In this research, the Chinese group was the most confident among the three. However, their confidence was justified by their high success rates. Thus, we cannot know whether Chinese undergraduates, such as those we sampled, have typically high confidence as a trait (e.g., Stankov et al. 2015) or whether their confidence is sensitive to their success in the task. Consistent with Ackerman (2023), the most overconfident group in our study was the Western sample, which also had the lowest success rate. These results are in line with the generally robust finding that poor performers tend to be the most overconfident (Dunning et al. 2003). Only scant literature addresses confidence and overconfidence in the Israeli population and previous findings are mixed (Hagendorff et al. 2021; Koellinger et al. 2007; Lundeberg et al. 2000). In the present study, the Israelis were the least overconfident group. The metacognitive approach asserts that overconfidence affects effort regulation, in that people who think they are performing well stop investing effort into trying to improve their achievement (e.g., Ackerman and Goldsmith 2011; see Fiedler et al. 2019 for a review). Thus, we call for future research to delve further into the attributes that make people overconfident and how this misplaced confidence is associated with their effort regulation as these are at the core elements of understanding thinking efficiency in the face of challenges.

We found that resolution was the only item-related dependent variable that differed between all three groups, with the Chinese group having the highest resolution, the Israeli group following, and the Western sample having the weakest resolution; although, it was still robust. As mentioned above, in reading-comprehension tasks, improved resolution was associated with in-depth processing (e.g., de Bruin et al. 2011). In terms of problem solving and perceptual decision making, in contrast, resolution has thus far been found to be resistant to manipulations aimed at improving sensitivity to misleading cues (Ackerman 2023; Haddara and Rahnev 2022). This resistance to improvement was also persistent across samples from Western and Israeli populations, such as those used in the present study, despite the substantial success differences between them (Ackerman 2023). The cultural sensitivity of resolution in problem solving found in the present study may inspire future research to discover what situational and cultural factors affect resolution; this would hopefully galvanize the development of means for improving it, as has been conducted effectively with reading-comprehension tasks (see Prinz et al. 2020). Improved resolution, in turn, carries hope for bettering both success rates and effort regulation as it supports more effective decisions about how to proceed after the solving session. For instance, good resolution is the basis for choosing which answers to withhold in order to improve success rates when an opt-out option is permitted (see Goldsmith 2016 for a review).

Improved monitoring accuracy may be considered also in terms of the effort invested in metacognitive monitoring itself. One consideration is the potential reactivity of collecting metacognitive judgments on the basic cognitive processes involved in performing the main task at hand. For instance, in memory research, there are consistent findings that collecting judgments of learning improves success in remembering related word pairs (e.g., Sky—Kite) but not unrelated ones (e.g., Sky—Vase; see Halamish and Undorf 2023 for a review). In reasoning and problem solving, in contrast, the results have been less consistent (e.g., Double and Birney 2017; Thompson et al. 2013). It is possible that the Chinese participants in the present study invested more effort in making their confidence judgments. This could have influenced both their success, as suggested by the reactivity literature, and their monitoring accuracy (see Coutinho et al. 2015; Maniscalco and Lau 2015). Comparing results for groups who are vs. are not asked to reflect on their performance across cultures may provide new insights into the issue of reactivity in the context of metareasoning research. Furthermore, Hoch et al. (2023) compared metacognitive judgments of effort to judgments of difficulty and confidence in several problem-solving tasks, including the MTT. Interestingly, effort and difficulty judgments were more strongly associated with the time invested in each task item while confidence was more strongly associated with success in those items. It is possible that these judgments reflect differences in reactivity as people seem to use them to reflect differently on the time invested and the success.

Effort regulation was analyzed in this study by employing the DCM (Ackerman 2014). This analysis allows for looking at effort regulation in terms of the strategic setting and adherence to stopping rules: the diminishing confidence criterion and the time limit. Looking at the continuum for confidence ratings reveals that the Israeli group invested more time than the Western group only in the items in which they had the least confidence. However, the Israeli group did not exceed the time they invested in items at the lower end of their confidence range for any higher levels of confidence. This is the typical pattern found under the DCM. The slight increase in the time invested in the hardest items is a classic reflection of high motivation to succeed, similar to the pattern shown with items assigned high incentives relative to those with lower incentives (Ackerman 2014; Undorf and Ackerman 2017). Studies dealing with the differential effects of incentives to succeed (e.g., Murphy et al. 2023) rarely look into their effects on various experienced difficulty levels. We hope that our study will inspire detailed analyses in such contexts, as well as in other cross-group comparisons (e.g., older and younger adults, Murphy and Castel 2022).

Looking at the spread of response times and confidence levels for the Chinese group relative to the other groups reveals both the main effect of longer time investment throughout the task and the unique increase in time invested in items at the middle scale of their confidence levels. In terms of the DCM (time prediction by confidence), this group’s pattern of effort regulation is unique. So far, prior analyses by the DCM have shown that people adhere to their time limit throughout the lower confidence levels (Ackerman and Levontin, forthcoming; Ackerman et al. 2020a; Undorf and Ackerman 2017). Investing relatively less time in items where people acknowledge a very low chance of success relative to their own mid-range confidence items—a pattern displayed only by the Chinese group in the present study—was suggested by Metcalfe and Kornell (2005) to be an effective strategy that reduces time investment in items which the person has no clue how to solve. Put differently, ending up with very low confidence reflects an acknowledgment of having wasted one’s time. Nevertheless, we see for all three groups that most solving time was invested in such items. This efficiency deficit has been termed *labor in vain* since the early years of metacognitive research (Nelson and Leonesio 1988) and has been seen as contradicting the notion that people are typically cognitive misers (see Ackerman et al. 2020b; Stanovich 2018 for reviews). The present study provides a cross-cultural view of labor in vain and cognitive miserliness. The results clearly show that the Chinese participants were motivated to invest a lot of time, despite having no particular incentive to do so.

However, beyond just determination to succeed, the present study exposes sophisticated effort regulation by our Chinese participants, with sensitivity to item characteristics. Specifically, they cut down on wasted time, giving up more quickly on some items (those that ended with very low confidence) and investing longer in others (those that ended with intermediate levels of confidence) relative to the other two groups. Although our analysis based on the size of the surrounding area sheds some light on this strategic allocation of thinking efforts, it does not seem to tell the entire story. Of course, these items may be idiosyncratically difficult, rather than having a common source of difficulty for all participants within a given culture. Clearly, further examination of this possibility is required.

Interestingly, given their determination to succeed and their self-reports of both having the least experience with thought-provoking games and being the least well-organized in terms of time management, we would expect the Chinese students to experience the task as more challenging than others. However, in the self-reports provided after performing the entire task, they rated the task as less challenging than did the other two groups. The effort regulation pattern seen in this group does chime with the fact that this group reported the highest need for cognition—i.e., liking cognitive challenges. In terms of mindset regarding intelligence, the Chinese group rated themselves as tending the most toward a fixed mindset, suggested by the mindset literature to indicate a preference toward avoiding mental challenges (Dweck 2008). In metacognitive research, both Miele et al. (2011), in learning, and Bae et al. (2021), in problem solving, found more strategic effort regulation among those with a growth mindset over those with a fixed mindset. Our findings stand out from these prior findings, suggesting that the need for cognition supersedes the fixed mindset among our Chinese participants. Another possible explanation for this pattern is that the cultural baseline for these scales differs between our samples—i.e., that the scales capture different attitudes in our Chinese and Western participants. Huang and Xie (2021), based on a large-scale survey of more than 33,000 Chinese citizens with a broad age range drawn from across the country, reported that a large majority tended towards a growth mindset rather than a fixed mindset. Cross-cultural differences in the interpretation of the scales we used are beyond the scope of the present study but raise interesting questions for future research.

The findings indicating high motivation to solve the problems in the Chinese sample are in line with evidence from qualitative studies in anthropology, sociology, and education (Fong 2004; Kipnis 2019; Li 2012). In particular, according to Confucianism, the most important purpose of human life is to “self-perfect” (*ziwo yuanshan*) or “self-cultivate” (*xiushen*). This notion of self-perfection is perceived as the core purpose of learning and intellectual endeavors. In addition, by this thinking, people are not born with the ability to self-perfect but must learn to do so. In this sense, success is derived not only from completing a task but from investing time in order to complete the task (Li 2012). However, other factors that might explain Chinese students’ labor in vain are the socio-cultural characteristics of China’s education system. This system is highly competitive, with significant emphasis placed on high-stakes standardized examinations; there is consequent pressure on schools, parents, and students to focus entirely on test scores (Zhao et al. 2015). The structure of this system is, almost by definition, designed to favor a prevention focus over a promotion focus, as discussed earlier. Our study reveals implications for efficiency compromises that should be further considered in future research.

The present study is unique in using a task that requires focusing on details over holistic perception. Indeed, we found that compact silhouettes, with smaller surrounding areas, were harder to solve than those spread over larger surrounding areas. For practical reasons (e.g., educational interventions), it is informative to understand effort regulation patterns within different cultures. Our analysis based on the surrounding area exposed the strong determination of the Chinese group in the face of the challenge of overcoming the interference of holistic processing. They invested most of their time in solving the most challenging items and, indeed, succeeded more than the other groups at all difficulty levels.

Our analysis of the cultural differences between the examined cultures focused on obtaining a theoretical understanding of factors affecting effort regulation. However, this analysis also furnishes practical insights that we hope will inspire the development of interventions. First, it is important for organizations to acknowledge differences among employees and customers from different cultures in their determination to successfully perform complex tasks. For instance, software developed to help in the performance of complex computer-aided tasks (e.g., medical diagnoses, engineering tasks) might be more or less effective depending on differences in the potential users’ determination to succeed in the tasks they perform. Usability testing might draw insights from the present study to enrich investigations of potential biasing factors that may generate illusions of success, other factors that may lead users to give up on using a software tool, and still, others that might encourage effortful thought, potentially leading to improved success and monitoring accuracy (see Ackerman et al. 2016). Furthermore, educational designers might use our findings as starting points for identifying aspects of educational tools and materials that deserve interventions. In line with recent calls for incorporating insights from metacognitive research into educational design (e.g., de Bruin et al. 2020), the present study may guide efforts to improve efficiency, monitoring accuracy, determination to succeed, and the acquisition of learning skills, ultimately promoting academic success.

To sum up, the present study adopted the metareasoning approach to expose differences between cultures. The nonverbal problem-solving task we used is highly challenging while not requiring any specific background knowledge. The myriad of measures collected allows for delving into effort regulation in order to understand the thinking processes that lead to different outcomes. Yet, clearly, this study is a starting point. First, the results should be replicated. Second, the extent of the generalizability and boundary conditions should be examined with different populations and different tasks, including tasks that share characteristics with that used in the present study (e.g., static visual tasks, well-defined problem-solving tasks) and others that have not yet been examined in this level of detail (e.g., tasks incorporating video components, creative tasks). All told, beyond the cultural differences at the heart of this particular study, we see this study as a demonstration of how to delve into effort regulation when comparing tasks, environments, and populations in numerous other contexts.

## Figures and Tables

**Table 1:** Means (and SDs) for the three samples.

	Western General Public	Chinese Undergraduates	Israeli Undergraduates	One-Way ANOVA		
Measure	*N* = 77	*N* = 74	*N* = 143	*F*	*p*	*η*_p_^2^
Self-report responses						
Challenge posed by the task	5.74 ^b^ (0.97)	3.96 ^a^ (1.41)	5.63 ^b^ (1.03)	64.69	<.001	0.31
Experience with thought-provoking games	3.65 ^b^ (1.60)	2.62 ^a^ (1.57)	2.97 ^a^ (1.38)	9.36	<.001	0.06
Actively open-minded thinking	5.13 ^b^ (0.94)	4.75 ^a^ (0.99)	5.36 ^b^ (0.80)	11.59	<.001	0.07
Time management	4.71 ^b^ (1.27)	4.29 ^a^ (0.91)	5.21 ^c^ (1.27)	15.33	<.001	0.10
Need for cognition	4.15 ^a^ (1.11)	4.96 ^c^ (1.11)	4.50 ^b^ (0.89)	12.28	<.001	0.08
Mindset (high represents fixed)	3.44 ^a^ (1.15)	4.19 ^b^ (1.39)	3.69 ^a^ (1.35)	6.42	=.002	0.04
Objective and metacognitive measures						
Response time	25.6 ^a^ (14.79)	54.2 ^b^ (27.95)	31.4 ^a^ (15.32)	48.87	<.001	0.25
Success rate	43.4 ^a^ (16.13)	66.5 ^b^ (20.54)	49.2 ^a^ (17.59)	34.42	<.001	0.19
Efficiency	1.24 ^b^ (0.62)	0.87 ^a^ (0.44)	1.09 ^b^ (0.51)	9.52	<.001	0.06
Confidence	62.5 ^a^ (12.35)	81.6 ^b^ (12.81)	61.1 ^a^ (12.91)	68.82	<.001	0.32
Calibration	19.1 ^b^ (16.53)	15.2 ^ab^ (18.25)	11.9 ^a^ (14.66)	4.3	=.008	0.03
Resolution	0.31 ^a^ (0.28)	0.57 ^c^ (0.34)	0.43 ^b^ (0.33)	13.68	<.001	0.09
Interference of holistic processing						
Surrounding area—Success	0.19 ^ab^ (0.26)	0.28 ^b^ (0.26)	0.18 ^a^ (0.27)	3.71	=.026	0.03
Surrounding area—Response time	0.03 ^a^ (0.22)	−0.14 ^b^ (0.22)	−0.01 ^a^ (0.23)	12.16	<.001	0.08

*Note*. Within each measure, means with different superscripts differ at *p* < .05, according to Tukey post hoc tests. As some of the measures were not normally distributed for at least one group, we performed the Kruskal–Wallis test parallel to the t-tests and obtained the same significant differences in all measures.

**Table 2:** Fixed-effects regression estimates (standard errors) across the groups.

Effect	Western General Public	Chinese Undergraduates	Israeli Undergraduates
Confidence	−0.80 *** (0.07)	−1.05 *** (0.11)	−0.70 *** (0.06)
Confidence^2^	−0.60 *** (0.20)	−1.57 *** (0.32)	−0.70 *** (0.17)

*Note*. *** indicates slope or curvature significance at the level of *p* < .001. Confidence^2^—Confidence squared.

## Data Availability

The study data set is available at: https://osf.io/wn38u/files/osfstorage (accessed on 21 August 2023).
